# Whole-Process Treatment of Combined Small Cell Lung Cancer Initially Diagnosed as “Lung Squamous Cell Carcinoma”: A Case Report and Review of the Literature

**DOI:** 10.3389/fimmu.2022.831698

**Published:** 2022-03-02

**Authors:** Yong Dong, Qijun Li, Da Li, Yong Fang, Chongwei Wang

**Affiliations:** ^1^ Department of Medical Oncology, Sir Run Run Shaw Hospital, College of Medicine, Zhejiang University, Hangzhou, China; ^2^ Department of Pathology, Sir Run Run Shaw Hospital, College of Medicine, Zhejiang University, Hangzhou, China

**Keywords:** c-SCLC, heterogeneity, neoadjuvant immunotherapy, NGS, case report

## Abstract

The rarity and complexity of histology lead to the low diagnosis rate and high misdiagnosis rate of combined small cell lung cancer (C-SCLC). Nowadays, C-SCLC has no commonly recommended therapeutic regimen, mainly conforming to SCLC treatment. Here, we report a patient initially diagnosed as IIIA “lung squamous cell carcinoma” by a small specimen. Radical resection was achieved after neoadjuvant immunochemotherapy, and the final surgical pathology was C-SCLC containing three different histological components. Moreover, in the literature review, we explored the therapeutic effect of neoadjuvant immunotherapy in C-SCLC, expounded the therapeutic conflicts among heterogeneous components, and analyzed the pathology complexity at the tissue, cell, and molecule levels in-depth, including possible genetic characteristics, origin, and evolution by next-generation sequencing (NGS).

## Introduction

The World Health Organization (WHO) defines combined small cell lung cancer (C-SCLC) as small cell lung cancer (SCLC) with any other histology of non-small cell lung cancer (NSCLC), like squamous cell carcinoma (SCC), adenocarcinoma, large cell neuroendocrine carcinoma (LCNEC), even spindle cell carcinoma and giant cell carcinoma. The mixed components can be one or more, and SCC is the most common type. C-SCLC’s low diagnosis and high misdiagnosis rate are related to the limitation of specimens, mainly taken by bronchoscope and aspiration biopsy rather than operation and autopsy. Therefore, as a relatively rare type of SCLC, the incidence of C-SCLC has not been accurately statistically analyzed (1). In earlier studies based on a small specimen, C-SCLC accounted for 2% of SCLC (2, 3), but it can reach more than 25% of surgically resected SCLC (4, 5). The detection rate of C-SCLC is affected by the size, number, integrity, and approach of biopsy specimens, as well as pathological analysis techniques. More than 90% of SCLC can be confirmed pathologically by small biopsies or cytological specimens under an optical microscope; however, most cases are not early-stage diseases and lose the opportunity of surgery (6, 7). As a result, SCLC cases rarely exist in large biopsies or operations (8). Patients with C-SCLC with different histology do not have a standard treatment scheme and are mainly treated according to the SCLC strategy. However, various NSCLC components of C-SCLC can affect the prognosis to some degree. The diverse non-small cell parts will add more complicated biological, clinical, molecular, and pathological to pure SCLC (P-SCLC) (8). Overall, depending on the extent of surgical resection and lymph node dissection, the prognosis of C-SCLC is generally better than P-SCLC (9). Tumors with non-small cell components show stronger resistance to radiotherapy and chemotherapy than P-SCLC.Cisplatin&etoposide/carboplatin&etoposide (EC/EP) regimen is still the first-line treatment with the best clinical benefits for C-SCLC; the application of three-drug combined regimens to C-SCLC still needs further exploration. This situation emphasized personalized and comprehensive treatment, including chemotherapy, surgery, radiotherapy, targeted therapy, and immunotherapy. Here, we report the detailed whole-process treatment of a stage IIIA lung cancer with three different histological components, which was initially misdiagnosed as pure lung squamous cell carcinoma (LUSC or LSCC). After neoadjuvant immunochemotherapy, the tumor was R0 resected, and the diagnosis was revised as C-SCLC. The latest evaluation indicated a radical cure reached after comprehensive treatments with adjuvant radiotherapy and immunochemotherapy. In addition, we also reviewed the clinical and molecular pathological features of C-SCLC, and the role of neoadjuvant immunotherapy in C-SCLC. Written informed consent was obtained from the patient for publication of the case details and accompanying images.

## Case Presentation

**Figure 1:**
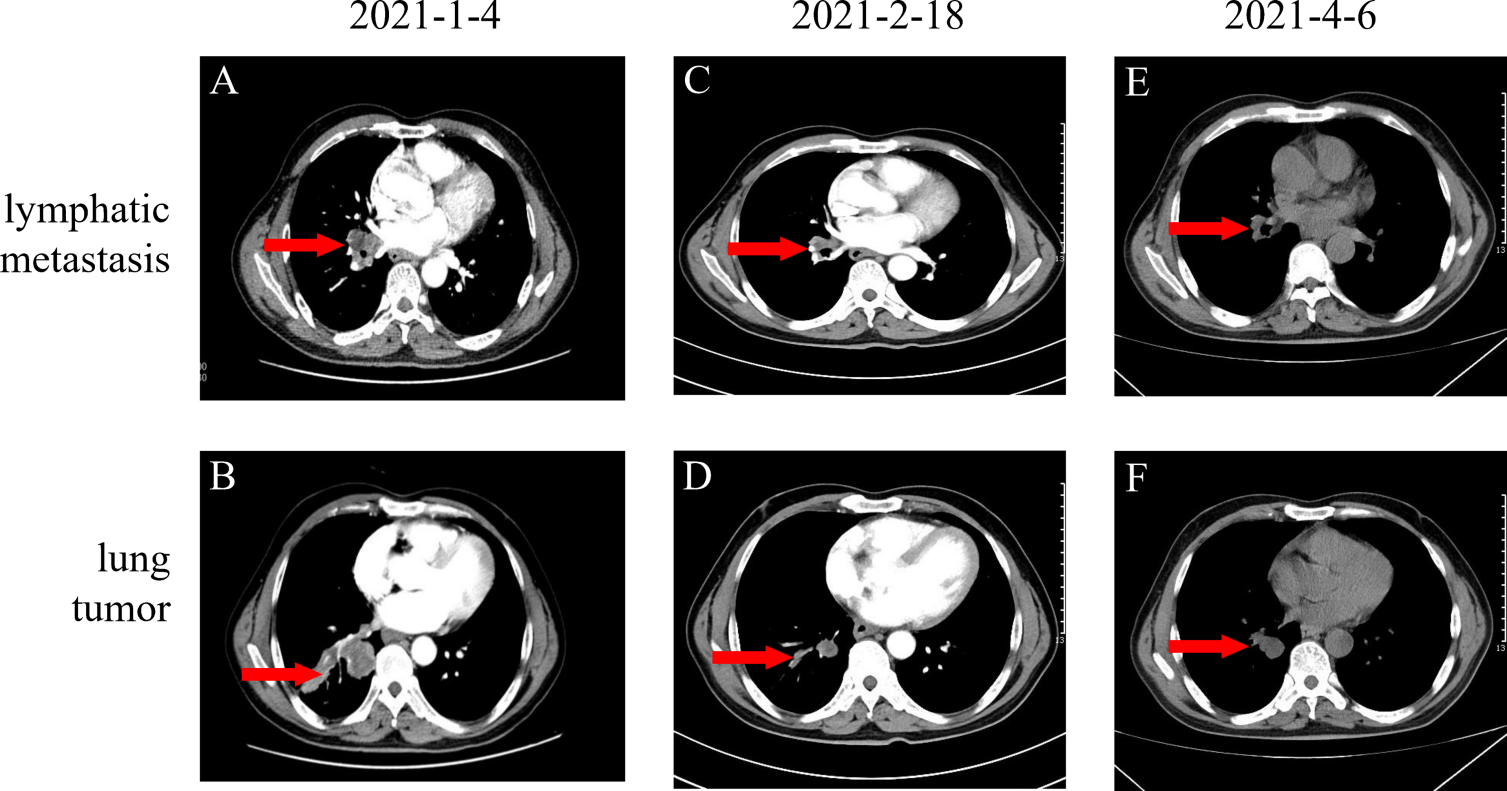
**(A, B)** Baseline images of CT before neoadjuvant therapy; **(C, D)** Original lung mass and lymphatic metastasis partial response (PR) after two cycles of AP (Albumin paclitaxel 400mg d1 and cis-platinum 60mg d1-d2) and Camrelizumab 200mg d1 **(E, F)** Original lung mass and lymphatic metastasis stable disease (SD) after four cycles of neoadjuvant therapy totally.

**Figure 2:**
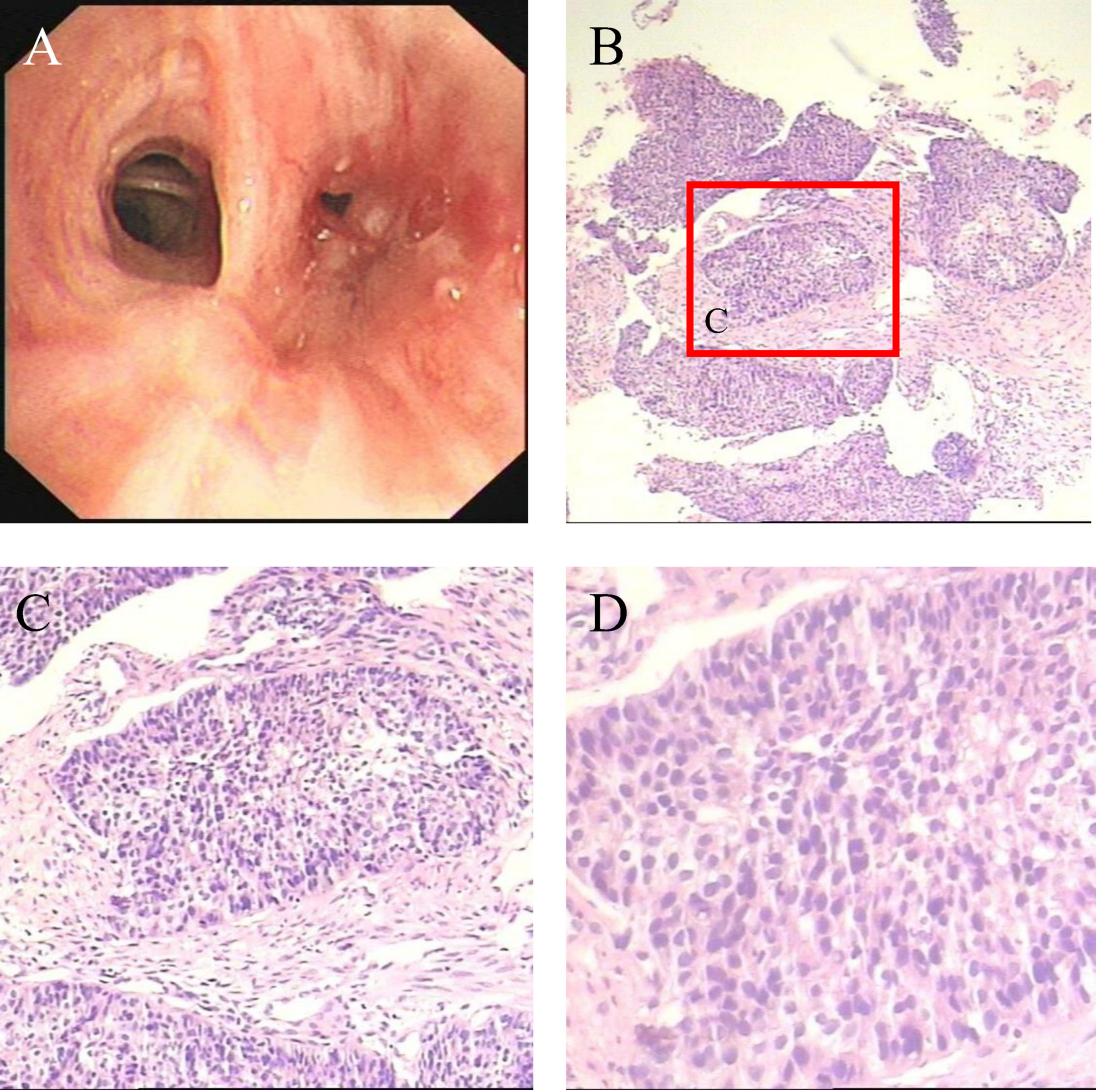
**(A)** Bronchoscopy demonstrates neogenesis in the basal segment of the right lower lobe **(B–D)** Microscopic appearances of the pulmonary tumor with H&E staining which show squamous cell carcinoma.

In December 2020, a 57-year-old man was referred to the Pinghu First People’s Hospital due to repeated expectoration for one year, which became severe in the latest week. Chest computed tomography (CT) indicated a right lung malignant tumor with right hilar lymph node metastasis, so he went to Sir Runrunu Shao hospital for further examinations. The patient had a 40-year smoking history of 10 to 20 cigarettes per day (400 to 800 package-year). Palpation of superficial lymph nodes was negative. The blood chemistry test were unremarkable, the levels of carcinoembryonic antigen (CEA), SCC and CA211 were 6.43 ng/ml (normal range, 0–5 ng/ml), 3.7 ng/ml (normal range, 0-1.5 ng/ml) and 4.89 ng/ml (normal range, 0-3.3 ng/ml), respectively. On Jan, 1^st^, 2021, Chest CT demonstrated a tumor measuring 3.2 cm in diameter in the right lower lobe with obstructive pneumonia and invasion to the adjacent basal segmental bronchus (**Figures 1A, B**). The tumor was possibly infiltrated into the visceral pleura, and enlarged lymph nodes of the right hilar were observed, which showed increased FDG metabolism in PET-CT and were considered metastasis. At that time, there were no obvious signs of distant metastasis. The bronchoscopic biopsy of the right lower lobe further confirmed that the pathology diagnosis was lung squamous cell carcinoma (LSUC) (**Figure 2**). The comprehensive evaluation showed the patient was in the advanced stage (cT3N1M0, stage IIIA), but the tumor still had a chance of complete resection. After a multidisciplinary team discussion, we performed preoperative neoadjuvant therapy to decrease the tumor burden and restage the disease. From 5^th^, Jan 2021 to 17^th^, Mar 2021, the patient received four cycles of immunochemotherapy: camrelizumab 200mg ig d1, albumin paclitaxel 400mg ig d1, cisplatin 60mg ig d1-2/q3w. After two cycles of treatment, the chest CT on February 18th, 2021, showed the mass was significantly smaller compared with the baseline CT (4^th^, Jan 2021) and achieved a partial remission (PR) defined by Response Evaluation Criteria in Solid Tumors (**Figures 1C, D**). However, chest CT on 6^th^, Apr 2021 showed the mass was slightly larger than two-cycle treatments before, and the lymph nodes were similar (**Figures 1E, F**), which was evaluated as stable disease (SD). We suspected that the tumor was rapidly resistant to that regimen, so right lower lobectomy combined mediastinal lymph node dissection *via* video-assisted thoracoscopic surgery (VATS) was performed on April, 8^th^ 2021.

**Figure 3:**
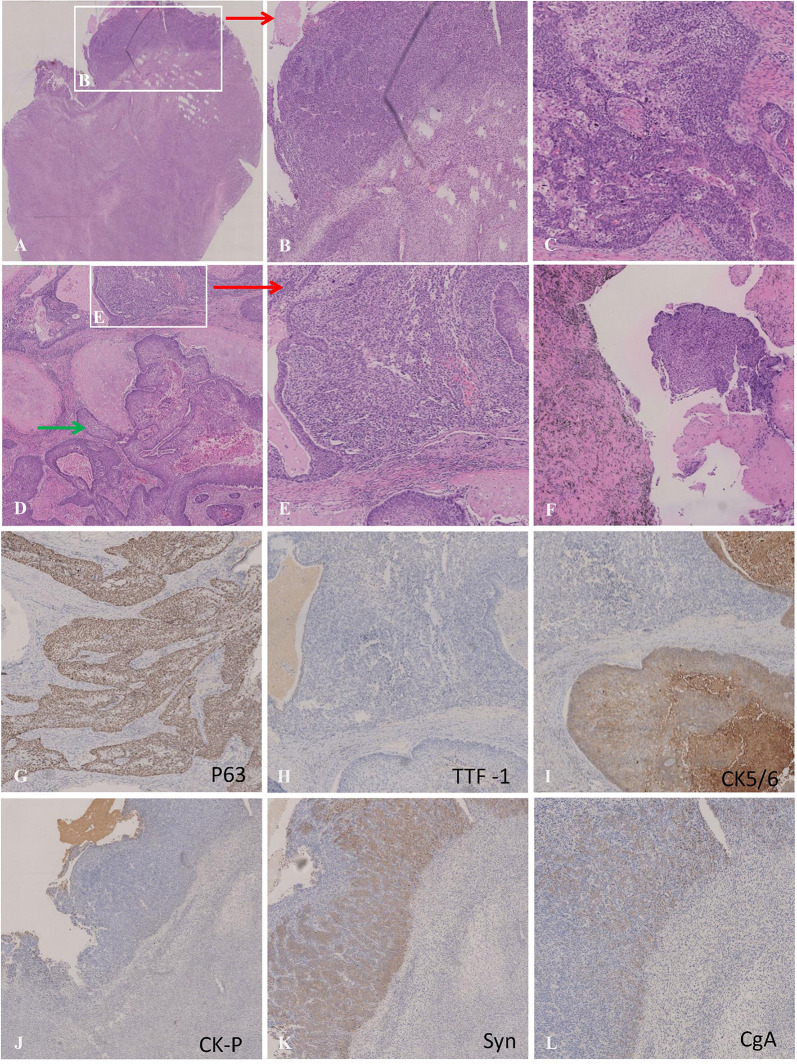
**(A)** Microscopic appearances of small cell carcinoma (upper section) :the cells were distributed in solid sheets, arranged like nests, beams, palisades and there were abundant blood vessels between nests. The cells were small, round or polygonal, with high nucleo-plasma ratio and bare nuclei. **(B)** Microscopic appearances of carcinosarcoma (lower section):tumor cells diffuse in sheets and cords,which were fusiform, with abundant cytoplasmand megakaryons. **(C)** Microscopic appearances of squamous cell carcinoma;:The cells were large in the shape of nests and eosinophilic red staining was observed in the cytoplasm. Intercellular Bridges were observed, and the fiber reaction between the nests was obvious. **(D)** Green arrow indicates squamous cell carcinoma invading cartilage (20×); **(E)** Carcinosarcoma; **(F)** Lymphatic metastasis with histopathology of squamous cell carcinoma; **(G–I)** Immunohistochemical staining for P63(+), TTF-1(-) and CK5/6(+) were the optimal immunohistochemical markers for squamous cell carcinoma; **(J–L)** Immunohistochemical staining for CK-pan (-), Syn (+) and CgA(+) was the best immunohistochemical panel in differentiating SCC from NSCLC.

Intraoperative findings revealed that right lung lobe partial adhesion to the chest wall with no pleural effusion or chest wall nodules. Gross pathology showed a mass of 2.5*1.9 cm in the right lung lobe and enlarged lymph nodes in the hilar, interlobar, and paratracheal. The resection margin was 0.5 cm away from the bronchus and pulmonary membrane. The tumor infiltrated bronchial cartilage and nerve. The histopathologic examination revealed three tissue components, containing squamous cell carcinoma (about 2*1cm), carcinosarcoma (about 1*0.9cm), and small cell carcinoma (about 0.7*0.2cm). In low power view, the HE stain showed the relatively separate small cell lung carcinoma area in the white blank in **Figure 3A**, where cells are round, small, high in nucleoid and plasma ratio, and organized in clusters under high power view (**Figure 3B**); IHC stains are consistent with SCC characteristics, including positive in synaptophysin (Syn) and CgA (**Figures 3K, L**). Another tissue section showed squamous cell lung carcinoma (**Figure 3C, D**) with cartilage involvement (Green arrow in **Figure 3D**). These big, rich-in-plasma cells formed clusters with necrosis inside and were positive for CK5/6 (**Figure 3G**), P63 (**Figure 3I**), P40, and negative in TTF-1 (**Figure 3H**); there was prominent fibrosis between clusters. The carcinosarcoma component was near SCLC (lower part of Figure A, white blank in **Figures 3D, E**), CK-P negative (**Figure 3J**), and Vimitin positive. Among the 21 lymph nodes in groups 2, 4, 7 to14, only one in group 11 confirmed squamous cell carcinoma metastasis (**Figure 3F**). The three components had no cross fusion or translational zone in HE and IHC stain images. In addition, the Ki-67 positive cells accounted for 80% in SCLC, and 40% in SCC, which could explain the tumor proliferation in the evaluation on 6^th^, Apr 2021. The PD-L1 (22C3) tests of the three histological components were all negative, and the TPS was less than 1%. Based on both the tumor size and lymph nodes metastasis, we restaged the disease for each histopathological component: the squamous cell lung carcinoma component was ypT3N1M0, stage IIIA, the small cell lung cancer component was ypT1aN0M0, stage IA, and the lung carcinosarcoma component was ypT1aN0M0, stage IA.

**Figure 4:**
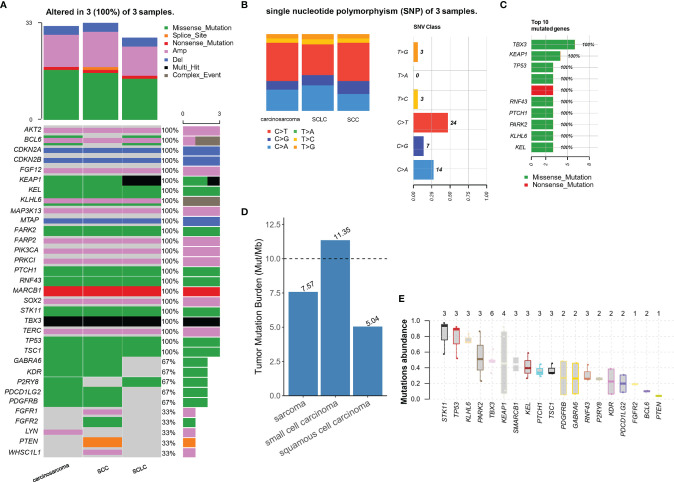
**(A)** All gene alteration in three types of tumor tissue; **(B, C)** describe the base mutation of three components and top 10 mutated genes of single nucleotide polymorphism (SNP); **(D)** The tumor mutation burden of three tumor **(E)** Mutation abundance of copy number alteration.

According to the surgical pathology findings and CT imaging, LUSC responded well to neoadjuvant immunochemotherapy; however, SCLC and carcinosarcoma did not shrink substantially. Gene tests revealed no EGFR, ALK, MET, and other treatable gene mutations in each pathology subtype. In addition, the TMB was 8muts/mb in lung carcinosarcoma, 11muts/mb in SCLC, and 5muts/mb in LUSC, respectively. The TMB, MSI, and mutation of NGS are shown in **Figure 4**. Therefore, considering the treatment response features of all malignant components, we finally chose a combination of postoperative immunochemotherapy and radiotherapy. LUSC was the main and the most advanced component of C-SCLC and was sensitive to neoadjuvant immunochemotherapy. The other two components were in a relatively earlier stage and not sensitive to preoperative treatment, which had been radically resected. Under these circumstances, we eventually started immunochemotherapy as adjuvant treatment from May, 8^th^ 2021: camrelizumab 200mg ig d1, albumin paclitaxel 400mg ig d1, cisplatin 60mg ig d1-2/q3w. In order to reduce the risk of radiation pneumonitis and immunotherapy-induced pneumonitis, camrelizumab was suspended to the second cycle (May, 28^th^ 2021). From June, 2^nd^ 2021, chest intensity modulated (IM) radiotherapy was implemented; the scheme was GTV (including the stump of the right lower bronchus) 6MV-X SAD 100DT 5992cGy/28f/38d, and CTV 6MV-X SAD100 DT 5040cGy/28f/38d. In the latest evaluation, the patient finished chest IM radiotherapy, and routine follow-up chest CT showed no tumor recurrence.

## Discussion

Most patients with unresectable NSCLC are treated with radical simultaneous or sequential radiochemotherapy, while the operation after induction radiochemotherapy is still conventional, though without first level recommendation. Accumulated evidence supported neoadjuvant therapy in selected patients with benefits from several aspects, such as prolonged survival and operation opportunity. ESPATUE trial showed that some unresectable stage III NSCLC became resectable after induction treatment. However, the results did not show more prolonged progression-free survival (PFS) or overall survival (OS) in the operation group; subgroup analysis showed that patients with T3N2 and T4N0-1 stage disease had significant long-term survival benefits (10). Many studies suggest that locally advanced NSCLC with resection potential can be restaged by neoadjuvant immunotherapy, which can lower recurrence risk, reduce tumor load, and increase the opportunity of R0 resection without increasing surgical complications (11). In addition, Immunochemotherapy can detect and clear micro-metastatic tumors. As for neoadjuvant treatment regimens, results from the NADIM trial and the ongoing phase II trial by Columbia University (NCT02716038) suggest that the pathological response rate of neoadjuvant immunochemotherapy is higher than neoadjuvant immune checkpoint inhibitor monotherapy (12). And until now, neoadjuvant immunochemotherapy has been more widely studied in phase III clinical trials of neoadjuvant immunotherapy (13, 14). Therefore, we finally chose a combined regimen of neoadjuvant immunochemotherapy.

Complete and precise diagnosis is the foundation of proposing comprehensive treatment strategies. In our case, the patient was initially diagnosed and treated as LUSC, but the postoperative histopathology revealed a C-SCLC with three different histological components. As a particular type of SCLC, the origin and biological characteristics of C-SCLC are still unclear. As formerly reported, the most common mix pathological types are large cell carcinoma, squamous cell carcinoma, and adenocarcinoma (5). Sarcomatoid or giant cell carcinoma are rarely involved, let alone small cell carcinoma and biphasic pulmonary tumors consisting of epithelial and mesenchymal components. Sümmermann E et al. reported a C-SCLC with a biphasic malignant tumor consisting of small cell carcinoma, adenocarcinoma, and fibrosarcoma in 2006 (15). In 2015, a patient with stage-IB C-SCLC (adenocarcinoma, squamous cell carcinoma, small cell carcinoma, and chondrosarcoma) underwent surgical resection without neoadjuvant therapy (16). Surgical resection could give us more tissue to analyze the origin and dynamic modification of C-SCLC components.

The possible origins of C-SCLC have been discussed in reviews and several case reports. First, the mutual transformation mechanism of NSCLC and SCLC. After the treatment of EGFR-TKI, the conversion of NSCLC to SCLC indicates plasticity between different types of tissue. Moreover, the increase of C-SCLC after neoadjuvant chemotherapy also supports (17) the residual components of non-small cell carcinoma could transform into small cell carcinoma. Second, different components of C-SCLC may have identical cell origins. Under different tumor microenvironments, the same tumor stem cells undergo the process of proliferation and differentiation; in the meantime, gene mutations or epigenetic changes lead to variations in biological behaviors, including metastasis, invasion, and drug resistance (18, 19). Besides conversion between components in one C-SCLC tumor, collision tumors refer to two or more independent primary tumors colliding or infiltrating with each other but no histologically intermediate differentiated cell clusters or mixtures (20). In our case, the three tumor components were separately distributed in pathological observation. So, we were more inclined to speculate that these three different histological components might derive from the same monoclonal stem cell. Unfortunately, there is only pathological evidence of LUSC before neoadjuvant therapy, and we cannot confirm whether carcinosarcoma and small cell carcinoma were original coexistence or treatment-induced. Previous studies suggested that SCLC with adenocarcinoma may originate from different clones (21), while carcinosarcoma or SCLC with squamous cell carcinoma may originate from monoclonal tumor cells (22, 23). However, there is no systematic study on the clonality of SCLC with biphasic tumors, and only one case report of SCLC with pulmonary blastoma and adenocarcinoma studied monoclonal origin through PCR-SSCP and the status of p53 (24). Highly differentiated NSCLC and pulmonary sarcomatoid carcinoma (PSC) often coexist in the same lesion, but a sarcomatoid component may branch out early from well-differentiated epithelioid components and accumulated mutations independently (25, 26).

To further explore the origin of each malignant subtype and to carry out targeted treatment for patients, we performed NGS of three tumor components after microdissection. Molecular subtypes detection plays an essential role in the comprehensive treatment of C-SCLC, since differences in histological types will lead to different biological and clinical characteristics. NGS can provide complete molecular information, identify therapeutic targets and discover new potential driving genes which may resolve the treatment dilemma. The mutation status of TP53, RB1, EGFR, Notch, ASCL1 can provide information on whether such components originate from a common ancestor or incidentally arise as collision cancers (27–30). Some epigenetic modifications, like histone acetylation, could be the secondary origin of NSCLC-related C-SCLC (31). In this case, we preliminary analyzed and found that three components had many identical mutations, particularly p53. At the molecular profile, these data indicate convergent mutation with a few subclone differences, so we considered that the complex histology is likely to originate from a typical multipotent stem cell, and p53 mutation occurred in the early stage of tumorigenesis before histologic differentiation. Subclone drifts cause subsequent acquisitions of other genetic mutations and finally form malignant tissues with similar molecular alterations but different characteristics. Although the NGS test suggested no treatable mutations such as EGFR, ALK, MET, ROS1, MET, BRAF, or KRAS, other possible meaningful mutations are detected. STK11 has the highest abundance of mutations among the mutation genes, followed by TP53 and KLHL6, and three mutations exist in each histological type.

Multiple biomarkers, such as PD-L1, TMB, and MSI, help us achieve more accurate predictions of the checkpoint inhibitors’ therapeutic response (32, 33). High TMB is defined differently through different detection methods and clinical trials. Some studies have shown that atezolizumab can prolong PFS in NSCLC patients with bTMB ≥ 16muts/mb (34). As for NSCLC patients, 6muts/mb of TMB is the recognized minimal value that shows a clinical response to ICIs (35–37). For SCLC, studies illustrate that patients with high TMB have a better clinical response to nivolumab or nivolumab plus ipilimumab, while no specifical TMB value is recommended for predicting treatment efficacy (38). The MSI of the three histological components was MSS type, and only the SCLC part of TMB exceeded 10muts/mb, which seems no beneficial immunotherapy and target therapy to this patient. However, there is no commonly recommended biomarker for SCLC to predict the efficacy of immunotherapy, and the high instability of genomic and chromosome in SCLC patients may theoretically be more sensitive to immunotherapy (39). There are several possible reasons. First, only PD-L1 inhibitors combined with chemotherapy has prospective benefit evidence in the first-line treatment of extended stage SCLC (ES-SCLC). In SCLC, application of PD-1 inhibitors, whether as first-line or back-line treatment or not, did not achieve the expected survival benefits. Secondly, the expression of PD-L1 in SCLC is low, which limit the predictive ability as the biomarker of classifying patients with potential benefits from PD-L1 inhibitors combined with chemotherapy. TMB has a proven predictive value in patients with recurrent SCLC, but its predictive value in SCLC patients with first-line therapy is not consistent with various studies (40). Therefore, PD-L1 and TMB cannot sufficiently predict the immunotherapy response in SCLC, but genotyping may provide new ideas for selecting the drug in the future. The key transcriptional regulatory factors classified SCLC into four subtypes (41). Different genotypes have different sensitivity to drug therapy and become a new biomarker for population screening beneficial to immunotherapy. For example, there is a significant synergistic effect between PARP or CHK1 inhibition and PD-1 inhibition in immunoreactive mouse models of SCLC-A subtypes (42). The gene expression profile of C-SCLC seems to be different from that of SCLC, and according to the results of NGS, we cannot match it with any subtypes. Therefore, further typing of C-SCLC as a particular type is needed to explore.

TP53 is active and vital in both PSC and small cell carcinoma, and the deletion of TP53 is necessary for the formation of SCLC (43). STK11 mutation is only found in biphasic PSC (44) and is a potential driver factor of PD-1/PD-L1 inhibitors resistance (45). KLHL6 is common in lymphoma, chronic lymphocytic leukemia (CLL), and other hematological tumors (46), and KLHL6/KLHL24-NTRK3 fusion was the first time found in LUSC recently (47). NTRK3 mutation is associated with enhancing immunity and immunogenicity in patients with lung adenocarcinoma (LUAD) and can predict a good prognosis in LUAD patients treated with ICIs (48). Among LUAD, PTEN mutation is often associated with the immune microenvironment and expresses differently in different TMB (49). NSD3 is a crucial regulator of LUSC and a potential driving factor in lung cancer with FGFR1 amplification (50), which may be a potential target for therapy. KEAP1 has been proved to be the driving factor of LUAD, and KEAP1/NFE2L2 is associated with drug resistance of chemotherapy and immunotherapy in NSCLC. A prognostic model constructed with five genomic mutations of CREBBP, KEAP1, RAF1, STK11, and TP53 showed that NSCLC patients with these five genomic mutations could not benefit from atezolizumab (51). Why the LUSC component was sensitive to immunotherapy, while SCLC and sarcoma were resistant to immunotherapy? The underlining biological behaviors are still unclear. According to a few studies of gene mutations in LUSC, patients was initially sensitive and then rapidly resistant to immunotherapy, which indicates that NSCLC and SCLC may not sustain their original genome or pheonotypes during immune system regulation.

The knowledge of C-SCLC is still lacking due to its low incidence and unsatisfied research evidence. Available conclusions are mainly based on retrospective analysis rather than multicenter prospective studies and large samples, and no sufficient evidence to identify the influencing factors of treatment and prognosis and clear consensus and guidelines on treatment. The clinical study of neoadjuvant immunochemotherapy in NSCLC is in full swing, while there is no related research in C-SCLC. According to the guidelines, if the patient is initially diagnosed with SCLC, he has no chance of neoadjuvant therapy or surgery. Moreover, the patient responded to neoadjuvant immunochemotherapy in the initial 2 cycles of treatment for the dominated LUSC component. The tumor grows slowly after 4 cycles of treatment, and we consider that the tumor heterogeneity could explain the overall resistance. Both imaging changes and tumor retraction in pathology illustrate that LUSC is sensitive to neoadjuvant immunochemotherapy, while SCLC and carcinosarcoma are resistant to this regimen. In this case, we accidentally explored C-SCLC treatment, and the whole-process treatment inspires us of the difference in biological behavior and treatment between C-SCLC and SCLC.

What’s more, the different components and their proportion of NSCLC in C-SCLC may affect the choice of treatment and prognosis. Neoadjuvant immunochemotherapy not only downstages the tumor but also enables us to obtain information on tumor drug sensitivity, which can guide the subsequent adjuvant therapy. Based on the patient’s response to immunochemotherapy, the proportion, and staging of each component, Referring to the scheme of adjuvant therapy in NSCLC, we chose the original immunochemotherapy regimen as postoperative adjuvant therapy and the same PD-1 inhibitor as maintenance therapy for one year. At the same time, considering the high malignancy and metastatic potential of carcinosarcoma and SCLC, even in the early stage, complicated components, and the clingy cutting edge of bronchus, the patient received prophylactic radiotherapy in the bronchial margin and lymph node area to decrease the risk of recurrence.

## Conclusion

Tumors have high heterogeneity and complexity. When the treatment response is not consistent with the expectation based on clinical evidence, it is reasonable to doubt that the biopsy specimens have not fully revealed all the histological types. Moreover, the histological components of C-SCLC may constantly be transforming during proliferation and treatment, which adds trouble to precise diagnosis and therapy. NGS provides a feasible method for treatment selection according to molecular information and clonal origin. This case report provides an example of a C-SCLC patient who benefited from neoadjuvant immunochemotherapy which gives C-SCLC patients more opportunities for surgery resection, pathology studies, and drug sensitivity tests, all of which relate to a better prognosis. Complete histological specimens can be conducive to diagnosing and precise treatment of C-SCLC and some unexplained drug resistance. In addition, the application of molecular biology is encouraged to study the origin, mechanism, tumorigenesis, treatment and prognosis of C-SCLC, in particular the mechanism that whether the components of NSCLC in C-SCLC will affect the treatment.

## Data Availability Statement

The original contributions presented in the study are included in the article/supplementary material. Further inquiries can be directed to the corresponding authors.

## Ethics Statement

Written informed consent was obtained from the individual(s) for the publication of any potentially identifiable images or data included in this article.

## Author Contributions

All authors have contributed to the preparation of this manuscript. All authors contributed to the article and approved the submitted version.

## Conflict of Interest

The authors declare that the research was conducted in the absence of any commercial or financial relationships that could be construed as a potential conflict of interest.

## Publisher’s Note

All claims expressed in this article are solely those of the authors and do not necessarily represent those of their affiliated organizations, or those of the publisher, the editors and the reviewers. Any product that may be evaluated in this article, or claim that may be made by its manufacturer, is not guaranteed or endorsed by the publisher.
